# Pig organs in humans: a forum on xenotransplantation

**DOI:** 10.1093/nsr/nwae208

**Published:** 2024-06-14

**Authors:** Weijie Zhao

**Affiliations:** NSR news and science editor based in Beijing

## Abstract

What if one of your organs, say, the kidney, the heart or the liver, deteriorates completely, and you are not lucky enough to have a human organ for transplantation? Do you want to receive an organ from a pig? That is not a joke, but a subject some physicians and researchers are working on—a field of research known as ‘xenotransplantation’.In 2022, David Bennett, a 57-year-old patient with terminal heart disease, received a pig heart and survived for 2 months after the surgery. Another recipient, Lawrence Faucette, survived for 40 days after similar surgery in 2023. Then, in 2024, Rick Slayman, the world's first recipient of a pig kidney, passed away 2 months after the surgery. Rick was able to get out of the hospital during these 2 months and the transplant team said there is no indication that his sudden passing was the result of failure of the pig kidney graft. Rather, it was related to his heart disease. In China, a liver xenotransplantation from pig to a brain-dead patient was reported in 2024 and the transplanted liver survived in the human body for as long as 10 days.On April 2024, *National Science Review* organized a panel discussion on xenotransplantation, which was chaired by Prof. Liangxue Lai and involved six other experts in the field. The panelists had in-depth discussions on xenotransplantation, covering its history and the (i) gene editing of the donor pigs, (ii) immunosuppressive regimens required by the recipients, (iii) challenges in preclinical and clinical research, (iv) relevant ethical issues and (v) prospects for the future of xenotransplantation.

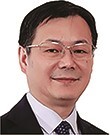

**Gang Chen**

Tongji Hospital, Tongji Medical College, Huazhong University of Science and Technology, Wuhan, China

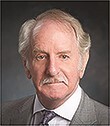

**David K.C. Cooper**

Massachusetts General Hospital, Harvard Medical School, Boston, USA

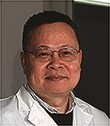

**Yifan Dai**

School of Basic Medical Sciences, Nanjing Medical University, Nanjing, China

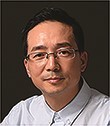

**Dengke Pan**

ClonOrgan Biotechnology Company, Neijiang, China

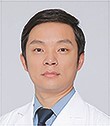

**Lin Wang**

Xijing Hospital, Air Force Medical University, Xi'an, China

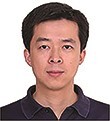

**Xuan Zhang**

Xijing Hospital, Air Force Medical University, Xi'an, China

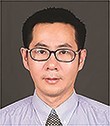

**Liangxue Lai (Chair)**

Guangzhou Institute of Biomedicine and Health, Chinese Academy of Sciences, Guangzhou, China

## Teaser

Seven panelists talk about xenotransplantation, covering its history, and the current status of (i) gene editing of the donor pigs, (ii) immunosuppressive regimens required by the recipients, (iii) challenges in preclinical and clinical research, (iv) ethical issues, and (v) prospects for the future.

**Lai:** Welcome to today's discussion on xenotransplantation. Firstly, let's introduce ourselves.

I am Liangxue Lai, currently working at the Guangzhou Institutes of Biomedicine and Health, Chinese Academy of Sciences. I have been conducting research in animal cloning, transgenic large animals and stem cells for >30 years. I made the world's first heterozygous *Gal*-knockout pig together with colleagues in Dr. Randall Prather's lab when I worked at the University of Missouri in 2002. As of now, my team has produced >100 types of genetically modified large animals, including pigs, dogs and rabbits, with important applications in biomedicine and agriculture.

**Cooper:** I am David Cooper. I was born in Britain and moved to the USA ∼40 years ago. I was trained as a cardiac surgeon and developed a special interest in heart transplantation. I was present at the first heart transplant ever carried out in the UK and I worked with Professor Christian Barnard, who did the first heart transplant anywhere, for several years in Cape Town.

After I worked in the USA for a few years, I decided to give up clinical work because my major interest had become xenotransplantation, as I realized that a lack of deceased human organ donors was a major problem. I have worked at four different centers in the USA, and now I am at the Massachusetts General Hospital. We have a fairly big research group in transplantation and I run a small group that is exploring pig kidney transplantation in baboons. I am also working with a group at the University of Southern California who are carrying out pig heart transplantation in baboons. These studies are initially being directed to pig heart transplantation as a temporary ‘bridge’ in babies who are born with complex congenital heart disease. The aim is to support the baby with a pig heart until a deceased human heart becomes available, which usually takes several months during which period many of the babies would likely die. We have carried out ‘bridging’ successfully in a baboon and we are optimistic this approach will prove valuable in the treatment of infants with complex congenital heart disease. The experience we gain from ‘bridging’ will enable us to progress to the transplantation of a pig heart as ‘destination’ therapy, i.e. it will support the baby for life.

I've been involved in xenotransplantation research since the middle 1980s—nearly 40 years now.

**Lai:** Thank you. I think Professor Cooper's career is a good reflection of the history of xenotransplantation.

**Dai:** I am Yifan Dai, currently at Nanjing Medical University. I started working on genetically modifying and cloning pigs in a company, PPL Therapeutics (now Revivicor), ∼25 years ago. During that period, we produced the first *Gal*-knockout pig, which was a milestone in the production of gene-edited pigs as sources of organs for xenotransplantation. Prof. Lai's group also achieved this important step at around the same time.

Then I worked with Dr. Cooper at the University of Pittsburgh for a few years. After that, I came back to China, where I am still working on the genetic modification of pigs for xenotransplantation and for other biomaterials or products.

**Chen:** I am Gang Chen, now working at Tongji Hospital of Huazhong University of Science and Technology in Wuhan. As early as 1998, when I was a PhD student, I was involved in probably the first xenotransplantation research in China—we used the human–DAF (decay-accelerating factor) transgenic pig to carry out pig-to-monkey xenotransplantation.

After that, I went to Canada, working with Dr. Robert Zhong on pig-to-baboon kidney xenotransplantation. Then I returned to China and engaged in both clinical and research work, mainly involving kidney transplantation. In recent years, I have worked with Dr. Dengke Pan and Dr. Yi Wang. We have performed >20 kidney transplants from genetically engineered pigs to rhesus monkeys.

**Pan:** I am Dengke Pan. I have been engaged in cloning pigs and genetically modifying them for >20 years. In 2010, I produced the first α-gal-knockout pig in China. I founded the company ClonOrgan Biotechnology in 2018. α-gal-knockout pigs have now been bred for >10 generations and, in addition, we have introduced >10 different gene modifications in our pigs for xenotransplantation. In recent years, we have used pigs with six gene modifications for preclinical and clinical research, including skin, kidney and liver xenotransplantation.

**Zhang:** I am Xuan Zhang, at Xijing Hospital of the Air Force Medical University in Xi'an. I am a member of Professor Kefeng Dou's group, which began research into xenotransplantation in 2013, first on the liver, and then extended to kidney, heart and other porcine tissues. Cooperating with Dr. Pan's company, pig liver xenotransplants have survived for 26 days. We are performing pig liver xenotransplantation in brain-dead human subjects (decedents) and are obtaining some encouraging results.

**Wang:** I am Lin Wang from the Department of Hepatobiliary Surgery of Xijing Hospital. I am currently the director of our department and also a member of Prof. Dou's team.

## A BRIEF INTRODUCTION

**Lai:** The first question of today's discussion is: why do we need xenotransplantation?

**Cooper:** That answer is fairly clear. It's because we do not have as many organs from deceased human donors as we need. In the USA, we have ∼40 000 organs of all sorts each year, but we have >100 000 patients waiting for organ transplants. We therefore need a new source of organs.

**Lai:** Would you please briefly introduce the history of xenotransplantation?

**Cooper:** The history goes back for quite a long time. We have seen mythological figures that are mixtures of organs. (For example, in the *Epic of Gilgamesh and Enkidu*, the monster Humbaba is described as a mixture of a bull, a lion, a vulture and a snake.) People have had the idea of mixing animal species together for thousands of years.

But, from a treatment point of view, it really started in the seventeenth century when they transfused blood from certain animal species into humans, commonly from sheep to patients who were psychologically disturbed. It was thought that, as sheep are very calm, their blood would calm the patient. Such treatment probably killed quite a few patients.

Then, organ xenotransplants began at the beginning of the twentieth century when surgeons knew nothing about the immune response. Xenotransplantation became less popular when allotransplantation was introduced. But, because of the shortage of allografts, in the mid-1980s, I and others began to consider using the pig as a possible source of organs.

The first relatively successful xenotransplantation surgeries were carried out in 1963 and 1964. Chimpanzee kidneys were transplanted into six human patients. The immunosuppressive therapy available at that time was very primitive and so most of the patients survived for only a few weeks, but one of them remained alive and well for 9 months and went back to work as a schoolteacher before she suddenly died (possibly from an acute electrolyte disturbance).

As time went by, it was concluded that carrying out organ transplants from non-human primates to humans was not satisfactory. That is why we've concentrated our attention for the last 30 or 40 years on pig organ transplants.

The first relatively successful xenotransplantation surgeries were carried out in 1963 and 1964. Chimpanzee kidneys were transplanted into six human patients.

—David K.C. Cooper

**Lai:** Why do we focus on pigs, but not other animals?

**Cooper:** There are a lot of good reasons why we don't want to use organs of non-human primates. First of all, the organs from most monkeys are much smaller than we would need. We would therefore have to use the ‘big monkeys’ such as chimpanzees or gorillas, but they are already endangered species, and there are not enough worldwide. Also, breeding them would be very time-consuming and expensive. For example, a baboon takes 9 years to reach its full size, and they only breed in ones or twos, whereas pigs can reach their full size within months and can breed in very large numbers.

Second, we don't know what microorganisms non-human primates might be carrying. We tend to know pig microorganisms because they have been bred in captivity for a long time. Chimpanzees and gorillas might carry something like the HIV virus. That's what people are worried about.

And I think there would be a big ethical outburst if we started slaughtering hundreds of thousands of chimpanzees because they're so similar to humans. The public would not like that. In contrast, in the USA, we kill more than 125 million pigs every year as food, and so we have become used to using pigs for our own purposes. Compared with goats and sheep, which are generally kept outdoors, pigs can be housed indoors, and so are easier to isolate from insects that might spread infection.

So there are good logistic and ethical reasons why we have concentrated attention on pigs as the potential source of organs for humans.

## XENOTRANSPLANTATION OF DIVERSE ORGANS

**Lai:** What are the organs that are suitable for xenotransplantation? Prof. Chen, maybe you can talk about this.

**Chen:** Kidneys and hearts are more suitable organs for xenotransplantation because, in preclinical studies in pig-to-non-human primate models, kidney transplants have been associated with the best results, followed by heart transplants.

The longest reported survival time for a kidney xenotransplant is 758 days and, recently, in a group of consecutive cases of kidney xenotransplantation, nearly all recipients survived for more than half a year. The longest recorded survival time for a heterotopic (auxiliary non-life-supporting) heart transplant was nearly 1000 days, while the longest survival for an orthotopic life-supporting transplant is to date 264 days. This is why heart and kidney xenotransplantation has been carried out in a small number of clinical cases.

**Lai:** How about the other organs, such as liver, lung and others?

**Chen:** At least currently, liver is unlikely to be used for human patients because, in preclinical studies, the longest survival has been <30 days.

**Cooper:** That is right, but there is still a great need for liver transplants. It may be that a pig liver will be used initially as a bridge to allotransplantation, or until the patient's own liver recovers, which can happen. In that case, we may not need as long a survival for the liver as we do for the heart and kidney. It may be sufficient for the pig liver to keep a patient alive for just a couple of weeks.

With the lung, we have poor results. It's very rare for a transplanted pig lung to function for even a week. There are problems that we don't see with the heart or the kidney. These may relate to the very fine structure of the lung, which can be easily damaged by antibody binding and/or complement activation, or because of coagulation dysregulation. But nobody can as yet identify exactly what the problem is.

There are also many other diseases that are amenable to treatment by xenotransplantation. For example, in the USA, there are 30 million patients with diabetes; if we can cure them with pig islet transplantation, that would be fantastic. Also, a group in Europe transplanted pig dopamine-producing cells into the brains of monkeys with an induced Parkinson's-like disease, and basically cured them. That is a very promising result.

We and others, including Yifan, have investigated pig red blood cell transfusion in primates. Eventually, we will have the right pigs as transfusion donors and solve the worldwide problem of insufficient human blood donors.

So, all of the pig organs might be suitable if we could get the technology correct. The potential for xenotransplantation is very considerable—from organs to cells.

**Pan:** I think pig skin and pig red blood cells may quickly come into clinical use.

**Zhang:** As Prof. Cooper said, in the short term, liver xenotransplantation can be used as a bridge treatment, keeping the patient

Kidneys and hearts are more suitable organs for xenotransplantation because, in preclinical studies … kidney transplants have been associated with the best results, followed by heart transplants.

—Gang Chen

alive before his or her own liver function recovers, or an allograft becomes available.

In the long term, the goal is to make liver xenotransplantation a destination therapy. However, the liver is a complex organ that can synthesize >500 proteins, and the proteins are not the same between pigs and humans. And so we need more fundamental research to achieve fully functional liver xenotransplantation.

**Cooper:** I'm very encouraged by Dr. Dou's research in a brain-dead subject. The pig liver graft survived for >10 days, which is pretty good for a liver, and a big step forward.

## THE IMMUNOSUPPRESSIVE REGIMEN

**Lai:** An important question is: how can we cope with the immune rejection response in xenotransplantation?

**Cooper:** The immune response to a pig organ is much stronger than the response to a human organ. It tends to be more dependent on antibody binding than on cellular rejection. Once rejection begins to develop, the graft can be lost within 24–48 hours, which is very unusual with a human graft, which takes a few days to be destroyed.

It's a major problem and, until very recently, we didn't know how to treat it. But it's become clear in the last few months that you can reverse rejection with a complement inhibitor, i.e. a drug that inhibits human or baboon complement from damaging the graft. I believe this is the most successful way of treating xenograft rejection but, if you don't start treating it very quickly, you lose the graft very rapidly.

**Chen:** If the donor pig is not genetically modified, the xenograft will face severe rejection mediated by the recipient's natural antibodies, resulting in rapid graft failure. But this ‘hyperacute’ rejection can be successfully prevented by using gene-editing techniques to knock out the main pig xenoantigens targeted by human (or primate) natural antibodies, and introducing several human ‘protective’ genes.

However, the xenograft may still suffer from acute or chronic antibody-mediated rejection, which may also lead to graft loss. Therefore, in addition to gene edits in the donor pig, the success of xenotransplantation also depends on the administration of a good immunosuppressive regimen. The inclusion in the regimen of an agent that blocks the CD40–CD154 co-stimulation pathway is important in preventing both cellular and antibody-mediated rejection. Additionally, we almost certainly also need anticoagulation and complement inhibition therapies.

The immunosuppressive intensity of this regimen is usually greater than that required in allotransplantation, and so the recipient is at greater risk of developing an infectious complication, which may threaten the life of the recipient.

**Cooper:** It is true that the immunosuppressive regimen needs to be stronger than that of allotransplantation, but it is certainly not as intensive as the regimen for patients who are having transplants in the presence of HLA (human leukocyte antigen) sensitization or ABO blood group incompatibility. I think the regimens we are using now are relatively safe.

## GENE EDITING IN PIGS

**Lai:** Now let's talk about the donor pigs. What should we do to the pigs to achieve long-term organ graft survival and function after xenotransplantation?

**Dai:** The current genetic modifications for donor pigs—three gene knockouts and insertion of three to six transgenes—have covered the major anti-rejection genes. I think that is good enough but, if we want to carry out more gene modifications, maybe we can add some protective genes such as hemeoxygenase-1 (HO-1) or PD-L1.

Regarding gene knockout, recently, we demonstrated that antigens of the pig major histocompatibility complex (MHC), also called swine leukocyte antigens (SLA), can stimulate human T-cell activation and induce the generation of anti-SLA antibodies that may cause antibody-mediated rejection after xenotransplantation. Therefore, SLA knockout should be considered to reduce the immunogenicity of the pig organ.

**Pan:** One point is that the expression level of human ‘protective’ genes in pigs should be relatively high—especially those for human complement inhibitors, such as CD55 or CD46, and those for anticoagulation factors, such as human thrombomodulin. Their expression needs to be similar to, or even higher than, that in humans. We have already achieved high expression of such genes in our six-gene-modified pigs, and this has been associated with long-term survival of these pig organs.

**Lai:** So far, my group has carried out >10 gene modifications to protect against immune rejection. As the gene-editing technologies have become so successful, I think maybe we can be more aggressive and modify more genes in the future. Maybe we can modify PD-L1, CDLA-4 or other genes to further solve the immune rejection problems.

If we carry out more modifications, it is possible that the pig will not survive, but we can use conditional gene-editing technology to deal with this issue. We can initiate gene expression in the pig organ just before the transplant and express some genes in specific tissue or organs (possibly for a finite period of time), so that the pig can survive. A germ-free clean environment can also help with the survival of the pigs.

The current genetic modifications—three gene knockouts and insertion of three to six transgenes—have covered the major anti-rejection genes.

—Yifan Dai

Maybe we can be more aggressive and modify more genes in the future.

—Liangxue Lai

If we keep adding genes, we may eventually be doing something detrimental rather than beneficial.

—David K.C. Cooper

Another direction is to express some humanized proteins in the donor pigs, so that the pig organs can be more functional after transplantation into human recipients. For example, we can express human serum albumin in the pig liver, human insulin in the pig pancreas or human renin in pig kidneys. Such modifications may help the organs to function adequately for a longer period of time.

**Pan:** I do not fully agree with you. I remember that Dr. Cooper once said in a talk that more gene modifications do not necessarily bring better results, and I suppose that's reasonable.

**Cooper:** The current gene editing may be sufficient, and there is a risk if you keep adding more genes, as we don't really know how they interact with other genes or whether they are all necessary. If we keep adding genes, we may eventually be doing something detrimental rather than beneficial. We should be careful about that.

**Dai:** That is why we need to test the function of the transgenes in animals. But unfortunately, the monkey seems not an ideal animal model.

**Cooper:** I think it's not possible to test each single genetic modification, but maybe we can theoretically predict which genes may be important and sufficient, and test organs from these pigs. For example, in a recent α-gal-knockout pig kidney xenotransplant in a decedent, the surgical team did not administer an anti-CD154 mAb-based regimen. The result should tell us whether α-gal-knockout and/or conventional immunosuppressive therapy is sufficient to prevent rejection.

## FROM PRECLINICAL RESEARCH TO CLINICAL PRACTICE

**Lai:** What are the major challenges for preclinical research on xenotransplantation?

**Pan:** In China, it's a great challenge to obtain suitable non-human primate animals as recipients. We use rhesus monkeys, which have higher anti-pig antibody levels than the specific-pathogen-free baboons used in the USA.

**Chen:** Yes. It is quite difficult to choose recipient monkeys that are suitable for the test. The natural antibody level is usually very high, and <10% of the monkeys can be selected.

**Cooper:** Whatever non-human primate you use, you have to select those with low antibody levels. One possible reason for the high level of antibodies in the monkeys used in China may be that their gastrointestinal tracts contain specific microorganisms against which a great deal of antibodies are produced, and these antibodies also bind to pig cells.

We are now using baboons from a facility in which they're housed under specific-pathogen-free conditions so that their antibody levels against pigs tend to be lower. These baboons are expensive, but I think it's worth it because you have a better chance of preventing rejection.

**Lai:** Maybe we also need a special facility in China.

**Cooper:** The non-human primate model has been extremely important in the last 20 or 30 years, but I think we're at a stage now at which we'll learn more from human clinical trials than we will from further studies in non-human primates, particularly because the pigs are genetically engineered for use in humans, not in monkeys. The pigs express human protective genes that are not as effective in baboons or monkeys as they will be in humans.

Another possibility is to use brain-dead human subjects as the recipient models. Dr. Dou's group has made valuable progress in this field, but I am not a great fan of the decedent model because there are pathological changes going on after brain death, e.g. an inflammatory response, metabolic incompatibility or metabolic instability, that make it a difficult model to manage. If you want to follow graft function for several months, it would be difficult to achieve this in the brain-dead model.

We'll learn more from human clinical trials than we will from further studies in non-human primates, particularly because the pigs are genetically engineered for use in humans, not in monkeys.

—David K.C. Cooper

**Lai:** What is the biggest challenge that has been met in the clinical trials?

**Cooper:** Patient selection is very important. A patient who is too debilitated probably will not recover. One of the two heart xenotransplantation patients at Maryland was too sick to benefit from the transplant. He had been on ECMO (extracorporeal membrane oxygenation) and hadn't got out of bed for several weeks before the xenotransplant. Even though the pig heart was functioning very well for 6 weeks, he was able to get out of bed only once during that time because he was so weak.

Currently in the USA, there are many patients who are waiting for a kidney transplant but will never get one. I suggest that it is better to offer them an alternative—a pig organ—than to withhold from them the reality that a human donor kidney will never come. We should pay more attention to the patients who might really benefit from xenotransplantation because there is no alternative for them.

**Lai:** Dr. Zhang, how did you persuade the family members of the brain-dead subject to accept the pig organ?

**Zhang:** In the recent liver xenotransplantation case, the patient was accidentally injured and was declared brain dead. His son is a medical graduate student, so, when we explained our xenotransplantation research to the family, the son quickly appreciated its meaning and the family decided to support the surgery. The son said that he hopes his father can be remembered as a special contributor to the development of medical science.

Regarding kidney transplantation, a difference between China and the USA is that, in the USA, many patients are excluded from the waiting list for reasons such as alcohol or drug addiction, so that their only chance is xenotransplantation. But, in China, we do not have similar restrictions. The first recipients in China may be those who have received human kidney transplantation more than once and are no longer acceptable for another, and who have been on dialysis for many years and so vascular access may now be impossible. A compassionate xenotransplant may be a reasonable choice for them.

The son [of the brain-dead subject] said that he hopes his father can be remembered as a special contributor to the development of medical science.

—Xuan Zhang

## DISEASE TRANSFER FROM PIG TO HUMAN?

**Lai:** One public concern about xenotransplantation is the risk of transferring animal infectious diseases to humans. Is it a real risk and can we prevent it?

**Chen:** We have found that pig cytomegalovirus (CMV) may cause donor-derived infection. In our recipient monkeys and the two brain-dead humans, we detected pig CMV not only in the transplanted xenografts, but also in other organs of the recipients, such as the liver, spleen, lung and heart. Although it is

In China, we established a DPF facility in 2022 and have obtained the first generations of virus-free pigs.

—Dengke Pan

uncertain whether a pig CMV infection can cause disease, I think it is still a risk. It is important to use pig donors that are CMV-negative because we do not have very effective drugs to treat it.

**Dai:** Most pig viruses can be excluded by breeding and housing the pigs in a designated-pathogen-free (DPF) facility, so I think it's not a big problem. In 2022, the first heart xenotransplantation recipient died with a pig CMV infection, and such incidents can be prevented in the future.

As for the porcine endogenous retrovirus (PERV) sequence within the pig genome, we are able to remove it by genetic modifications or breed pig lines with very low levels of PERV, so I think it is not a big issue, either.

**Cooper:** I agree with Yifan. With regard to PERV, which we cannot get rid of by keeping the pigs in a clean environment, there is currently no evidence that it can cause graft failure as CMV does. But I think that is the sort of question that the regulatory authorities will have to decide. Currently, the regulatory authorities in the USA do not seem to think it's essential to have a pig that is free of PERV.

Actually, the pigs from clean facilities will be ‘cleaner’ than human donors, so I don't think infection in xenotransplantation will be a bigger problem than that in allotransplantation. The real challenge is still the immune rejection responses.

**Pan:** In China, we established a DPF facility in 2022 and have obtained the first generations of virus-free pigs. Most viruses and bacteria are erased. I also agree that PERV is not a major problem and can be ‘knocked out’, if necessary.

**Dai:** Regarding the virus issue, sample monitoring is very important. That is why the US FDA requires researchers to maintain pig tissue and blood samples in the freezer for 50 years, so that we always can go back to check and identify hitherto ‘unknown’ infectious viruses or bacteria.

## ETHICAL CONCERNS

**Lai:** Will xenotransplantation break the species barrier between human and pig? That is a question that may be asked by many people.

**Cooper:** I think that, if we follow two principles, this concern can be addressed. First, we should not change the brain or the thinking process of either the pig or the human. Second, we should not modify the reproductive system so that we will not produce a child with pig genes or a piglet with human genes. Following these two principles, the fact that you have a pig organ in you is irrelevant because that pig organ is going to die when you die. So I don't think that's a problem, but I don't know what everybody else thinks.

**Chen:** Patients who receive a xenograft probably need to be closely observed and be subject to certain restrictions, such as limitations on fertility to prevent unexpected changes in the next generation.

**Lai:** So the question is whether the recipients of pig organs can be allowed to marry and have babies? What's your opinion?

**Dai:** I think they can be allowed to do so.

**Pan:** Yes. I have worked for many years on transgenic pigs, and I think there is little chance of transferring pig genes into a human body that will be passed on to the next generation.

**Cooper:** We also have to consider the psychological aspects of having a pig organ. Some people may feel uncomfortable and wonder how other people view them. It's particularly important with children, because children can be very unkind to other children. They can bully them and tease them if they know that the child has a pig heart.

**Lai:** Yes, and related to this, the next question is how to better communicate with the public?

**Dai:** Every time a news item on xenotransplantation appears in the media, it's a good chance to educate the public. The public is more positive than negative about xenotransplantation, and every opportunity to educate them should be taken.

**Cooper:** That is quite correct. When I worked in Alabama, we carried out some surveys of patients and their families. It was quite clear that many of them really didn't know what would be involved in xenotransplantation. But when we asked mothers of babies who were waiting for heart transplants if they would accept a pig organ for their baby, they generally said ‘if it will keep the baby alive, yes, I would accept it’. And when they were told that the pigs would be genetically engineered, they were pleased because they felt that the ordinary pig is a rather dirty beast, but a genetically engineered one is much better.

So, the public's knowledge of xenotransplantation is still very limited, but I think they'll get used to the idea if we can educate them properly. Actually, exactly the same happened 50 or 60 years ago with allotransplantation, when people didn't understand it and thought it was odd. Yet now, the majority of people accept it pretty well.

**Wang:** Besides communication with the public, communication with the authorities is also an essential issue. Take surgery on brain-dead subjects as an example. It was not easy for the leaders of our hospital, our university and the Air Force to

So the question is whether the recipients of pig organs can be allowed to marry and have babies?

—Liangxue Lai

understand what this would involve and to accept it. One of their major concerns was whether the public would accept the concept of xenotransplantation. The turning point appeared when the journal *Nature* interviewed us and posted a news report on this subject. The leaders read the report and began to accept our experiments. So I think, currently in China, there is still a long way to go before the public accepts the idea of xenotransplantation.

**Pan:** I have similar experiences. I used to communicate with the local government officials in the city of Neijiang. At first, they didn't understand xenotransplantation and considered it something of a joke. But, after 5 years, they now support our work enthusiastically. Dr. Cooper once reminded me that we should write more papers and actively communicate with local and national officials in order to introduce our new ideas to them, and I believe that approach has worked out well.

It was not easy for the leaders of our hospital, our university and the Air Force to understand what this would involve and to accept it.

—Lin Wang

## FUTURE OF THE TECHNOLOGY

**Lai:** What will be next news in this field within 5–10 years? What can we expect to happen in 20 or 50 years?

**Cooper:** I believe that, within a few years, we'll have pigs such that, when we transplant the organ, we need to give no immunosuppressive therapy at all. The organ will protect itself by the genetic engineering. That will be wonderful for the patients because they won't be immunosuppressed and will not be susceptible to infections—or at least the risk will be much lower.

In the long term, maybe in 50 years, I believe that there will be no need for deceased human organ donation because the pigs will provide better, cleaner and less infected organs than humans do. I suggest that, eventually, allotransplantation will be of historic interest only. In probably 50 years, people will be sitting around saying: ‘Do you know that once upon a time they transplanted organs from dead people? I can't believe they did that.’ Eventually xenotransplantation will be the only form of transplantation, unless there are technologies such as stem-cell-derived human organs that may supersede pig organs.

**Chen:** I expect that, in the future, xenotransplantation may coexist with allotransplantation for a long time. More and more patients will accept xenotransplantation, but it is unlikely to completely replace allotransplantation.

**Wang:** After our recent surgery, an expert in organoid research told me that, in her opinion, xenotransplantation is a much better way to treat the diseases of many different organs than the organoid. So I think xenotransplantation is perhaps one of the most promising techniques in this field.

I suggest that eventually allotransplantation will be of historic interest only … xenotransplantation will be the only form of transplantation.

—David K.C. Cooper

Xenotransplantation may coexist with allotransplantation for a long time … but it is unlikely to completely replace allotransplantation.

—Gang Chen

According to the plans of our group, in this year, we would like to do a whole-liver transplant from a pig into a brain-dead subject. In the future—maybe next year or sometime later—we would like to transplant a pig liver into a living patient with liver disease.

**Pan:** People often ask me when pig organ transplantation will become routine clinical practice in China or in the USA. Dr. Cooper, what do you think?

**Cooper:** I think we're already on the verge of it in the USA. We're doing one or two cases and, if we can show that these are relatively successful, the regulatory authority will likely be fairly positive about it. My impression is that the regulatory authorities have realized that it is going to occur and do not have major reservations about it anymore. So we just have to prove to them that we can achieve considerable success.

**Lai:** Thank you. Then let's go to the last question: will there be other technical choices? For example, will we be able to grow human organs in pigs?

Actually, my group has done some work on the humanized organ in pigs. We use human stem cells to produce chimeric embryos (between human and pig), and we have obtained mid-stage kidneys of ∼28 days. This is very preliminary work, and we still don't know when we will obtain mature and functional human organs in pigs. The gestation term of humans is ∼10 months, and that of pigs is ∼4 months. How to coordinate organ development is a big challenge. I think that, maybe in 5–10 years, we will be able to do something in the donor cell and recipient pig to synchronize development and get better results in this direction.

**Dai:** Prof. Lai's work on humanized organs is very promising. I think the two methods can develop side by side. On one side, we modify pig organs and, at the same time, we work on humanized organs. Especially for some organs, such as liver and lung (that are difficult for xenotransplantation), if we can make humanized livers and lungs available, it will be very good news for all the patients. But maybe we should try an ‘easy’ organ first and start with the kidney.

**Pan:** The humanized organ is chimeric, as it is difficult to achieve a humanized organ in pig body with 100% human cells. In that case, we should use the genetically modified low-immunogenicity pigs to make such organs, so that the immune rejection can be controlled at a relatively low level.

**Lai:** I think we can combine these two technologies to solve the immune rejection problem. Adding human cells to the pig organs may be able to solve some problems that cannot be solved directly by genetic modification of the pigs. I hope researchers in these two directions can work together to solve these issues.

**Cooper:** All of these approaches should be explored if we have the time and the funding to do it. But I think that, with genetic engineering technology, we will be able to produce pig organs that do not induce an immune response in the human recipient, or at least the grafts can be protected from the immune response. If that ‘universal donor’ comes into reality, any alternative approach is unlikely to be more successful. So, if we concentrate on genetic engineering of pigs, we’ll eventually solve all the problems. That's an extreme view, but I really believe that will be possible.

